# Predictors of Word and Pseudoword Reading in Languages with Different Orthographic Consistency

**DOI:** 10.1007/s10936-022-09893-5

**Published:** 2022-07-04

**Authors:** Maria-José González-Valenzuela, Dolores López-Montiel, Fatma Chebaani, Marta Cobos-Cali, Elisa Piedra-Martínez, Isaías Martin-Ruiz

**Affiliations:** 1 Department of Developmental and Educational Psychology, Faculty of Psychology and Speech Therapy, Univerisity of Málaga, Campus universitario de Teatinos s/n, 29071 Málaga, Spain; 2 Department of Psychobiology and Methodology of Behavioral Sciences, Faculty of Psychology and Speech Therapy, Univerisity of Málaga, Campus universitario de Teatinos s/n, 29071 Málaga, Spain; 3 Educational Sciences Department, The Higher Normal School of Kouba, Algiers, Cite Garidi 1 Bt “c” N°4 kouba. Alger, Vieux Kouba, Algeria; 4 Faculty of Philosophy, Letters and Educational Sciences, University of Azuay, Avenida 24 de Mayo, 7-77, Cuenca, Ecuador

**Keywords:** Cognitive predictors, Reading, Spanish, Arabic, Orthographic consistency

## Abstract

This study analyzes the impact of certain cognitive processes on word and pseudoword reading in languages with different orthographic consistency (Spanish and Arabic) in the first year of Primary Education. The study was conducted with a group of 113 pupils from Algeria and another group of 128 pupils from Ecuador, from a middle-class background and without any special education needs. The participants were assessed in terms of their reading ability of words and pseudowords, knowledge of letters, phonological awareness, rapid automatized naming, and phonological memory. Using a correlational design, descriptive-exploratory, bivariate, and hierarchical multivariate regressions were applied to the different measures of reading in each language. The findings show that knowledge of letters, phonological awareness, rapid automatized naming, and phonological memory contribute differently to the explanation of reading ability in each group at the start of compulsory schooling. These results have important implications for the teaching of reading skills and the prevention of specific learning disabilities, as well as the theory of reading acquisition.

## Introduction

The relationship between cognitive variables and reading has been the subject of research for many years now. Recently, there has been interest in studying at early ages the joint predictive relationship between some of these variables in languages with different orthographic consistency, to analyze their implicit processes and offer solutions for learning disabilities (Al-Tamimi & Rabab’Ah, 2007; Caravolas et al., 2013; Georgiou et al., 2008; González-Valenzuela et al., 2016).

There is consensus regarding the existence of different reading acquisition rates depending on orthographic consistency and that reading is acquired faster and earlier in more consistent languages compared to less consistent languages (Serrano et al., 2010; Seymor et al., 2003). However, the differences or similarities in the mechanisms in which the acquisition of reading is grounded depending on orthographic characteristics are unclear. Some studies support these differences in the cognitive processes involved in reading, but others do not (Caravolas, 2018; Frost, 2005; Joshi & McCardle, 2018; Ziegler & Goswami, 2005). The first are based on a dual-route model and defend the importance of grapheme-phoneme rules (phonological processing) in the reading of pseudowords in languages with consistent orthographies, along with the relevance of accessing visual-orthographic representations (visual processing) in the reading of words in less consistent orthographies (Georgiou et al., 2008; Moll et al., 2014; Tolchinsky et al., 2012; Ziegler & Goswami, 2005). The second are based on the model of self-teaching and argue that the orthographic representations formed in different languages are grounded in the repeated use of phonological representations and in grapheme-phoneme or phoneme-grapheme translation. Phonological processing or access to phonological representations, therefore, would be most relevant in different reading measures and in different languages (Share, 2008; Share & Shalev, 2004).

Despite the different opinions about visual and phonological processing in languages with different orthographic consistency, the majority of studies highlight the relationship between reading and certain variables, such as knowledge of letters, phonological awareness, rapid automatized naming, and phonological memory, among others (Al-Tamimi, & Rabab’Ah, 2007; Georgiou et al., 2008; Gómez-Velázquez et al., 2010; Tolchinsky et al., 2012). However, there seems to be no consensus about whether the role played by these variables in the explanation of reading is similar or different depending on orthographic consistency. Some studies argue that the influence of these variables on reading varies according to orthographic consistency, but they do not all agree on the role played by each of these variables in different languages (Furness & Samuelsson, 2009; Tibi & Kirby, 2018; Vaessen et al., 2010; Ziegler et al., 2010). Hence, some studies highlight that at early ages, for example, phonological awareness seems to be more relevant in more consistent languages, whereas rapid automatized naming seems to be more relevant in less consistent languages (Babayigit & Stainthorp, 2011; Georgiou et al., 2008; Landerl et al., 2019; Pittas, 2018). Other studies note that, as children get older and have more reading experience, phonological awareness becomes more relevant in less consistent languages, and rapid automatized naming is more relevant in more consistent languages (Georgiou et al., 2008; Nielsen & Juul, 2016; Vibulpatanavong & Evans, 2019). Furthermore, some studies argue that the relationship between some of these variables and reading seems to be similar in languages with different consistency, and some of them proffer a universal theory regarding the acquisition of reading (Caravolas et al., 2013; Frost, 2005; Furness & Samuelsson, 2009; Hassunah-Arafat et al., 2017; Ziegler & Goswami, 2005). From this perspective, in languages of different orthographic consistency, knowledge of the name and sound of the letters, phonological awareness, and rapid automatized naming are strong predictors of reading, whereas phonological memory displays a weaker relationship (Caravolas et al., 2012; Georgiou et al., 2008; Parrila et al., 2004; Vaessen et al., 2010). These findings about the poor correlation of phonological memory with reading ability might be due, on the one hand, to the way of measuring phonological memory, which in most cases is evaluated by means of verbal memory or digit memory tests, rather than evaluating it as the capacity for phonological recoding when accessing lexicon, which would explain how phonological information stored in the memory is retrieved. On the other hand, it could be due to whether phonological awareness and rapid automatized naming have been considered jointly or separately, since some studies argue that phonological memory only correlates with reading when these variables are considered jointly (Parrila et al., 2004; Suárez-Coalla et al., 2013), on account of the relationship between memory and phonological awareness (Wagner et al., 1997).

With regard to the Spanish language, some studies have analyzed the relationship between some of these variables and reading, but not many have conducted a joint analysis of the influence of these variables in Arabic or compared these influences in both languages with different orthographic consistency at the start of compulsory schooling. Translinguistic studies tend to compare European languages with one another (Caravolas et al., 2013; Georgiou et al., 2008; Moll et al., 2014; Patel et al., 2004). In this regard, predictors in Spanish, as a language with a high level of orthographic consistency, have been compared with other less consistent European languages (Caravolas et al., 2012, 2013; Tolchinsky et al., 2012), but not with Arabic.

The theory of granularity supports the idea that consistency in each language is relative since it depends on the orthographic systems compared (Carrillo et al., 2013). In other words, a language will be less orthographically consistent when the graphemes represent phonemes less precisely on account of their phonological structure (coarse grain), whereas it will be more consistent when the graphemes represent phonemes more precisely (fine grain) (Ziegler & Goswami, 2005). In this regard, Arabic would be classed as a less consistent language than Spanish. In Spanish, there are 27 letters (5 vowels and 22 consonants) that can be represented in upper or lower case. Syllables do not necessarily begin with a consonant, and there are different types of syllable structure (for example, CV, VC, VCV, CVC, CCV, CVV, CCVC, CVVC) (RAE, 2014). There is biunivocal correspondence between graphemes (letters) and phonemes (sounds) since most letters have a single sound, and each phoneme corresponds to a single grapheme, although there are some exceptions (for example, the letter j or g can be pronounced /j/). Furthermore, each grapheme only has one written form and is written in the same away regardless of the letters on either side, and all phonemes are written (with the exception of /h/). In Arabic, there are 28 letters (3 vowels that can be elongated, 23 consonants, and 2 semi-consonants), represented in lower case only. Syllables must start with a consonant and are dominated by vowel-consonant alternation, reducing syllable structures to CV, CVV, CVC, and in some cases CVVC (Baccouche, 2003; Cohen, 2007). Biunivocal correspondence between a grapheme and a phoneme is less frequent since there are many graphemes that are not pronounced, or which are pronounced differently to the way they are written. Furthermore, some letters have more than one written form depending on their placement within the word (beginning, middle, or end), following the linking rules with the preceding letter, and some have dots in different positions (above or beneath the letters) whereas others do not (Abu-Rabia & Taha, 2004; Abu-Rabia et al., 2003; Taha, 2013; Taha et al., 2013).

Furthermore, the impact of these cognitive variables on reading can also vary according to the reading measures considered (Aguilar-Villagrán et al., 2010; González-Valenzuela et al., 2016; Suárez-Coalla et al., 2013). Hence, phonological awareness correlates more closely with pseudoword reading than with word reading, whereas phonological memory corresponds more closely with word reading than pseudoword reading. This could partly be due to how children use reading processes. If a child performs well in word reading but makes many mistakes with the pseudowords, this could indicate that their reading is based on the lexical route and that they have not fully assimilated the rules of grapheme-phoneme conversion, possibly because they have not yet assimilated the orthographic representation of frequent words (Cuetos et al., 1996). Hence, the study presented here considers both reading measures—words and pseudowords—in both languages.

For these reasons, the aim of this paper is to analyze the impact of certain variables (knowledge of letters, phonological awareness, rapid automatized naming, and phonological memory) on word and pseudoword reading in languages with different orthographic consistency (Spanish and Arabic), at the age of six.

## Method

### Participants

The sample is made up of two groups of children, in the first year of Primary Education, with no special education needs, according to the reports drawn up by the school psychologists. These two groups belong to schools in Ecuador and Algeria, and they are all native speakers of Spanish and Arabic, respectively, who had begun learning to read in the final year of Early Years Education. Table 1 summarizes the socio-demographic characteristics of the sample: age of the children, gender, and parental level of education.

**Table 1:** Sample sociodemographic characteristics

Language group	Age in months*M* (*SD*)	Gender	Mother’s education level (%)			Father’s education level (%)		
			1 Low	2 Middle	3 High	1 Low	2 Middle	3 High
*Spanish* (*n* = 128)	81.05 (3.94)	78 F/50 M	0.8	60.2	35.2	0.8	59.4	35.2
*Arabic* (*n* = 113)	80.69 (4.30)	62 F/51 M	8	64.6	27.4	9.7	63.7	26.5

*M*   mean,* SD*  standard deviation, *F * female, *M * male, *Low* primary and pre-secondary studies, *Middle * junior high and high school (technical and non-technical), *High* college and graduate

The first group was made up of 128 Spanish-speaking pupils, from a school in Ecuador: 78 girls (60.9%) and 50 boys (39.1%), aged 6 (*M* = 81.05 months, *SD* = 3.94). The second group was made up of 113 Arabic-speaking pupils, from a school in Algeria: 62 girls (54.9%) and 51 boys (45.1%), aged 6 (*M* = 80.69 months, *SD* = 4.30). The schools were chosen at random from a middle-class sociocultural area, in both cases.

Parents in both groups had an intermediate level of education (*Mdn* = 2, *Mo* = 2). No association was found between intermediate and high levels of education, although an association was found between the low level of education of mothers [χ^2^ (2, *N* = 241) = 8.68, *p* < 0.05] and fathers [χ^2^ (2, *N* = 241) = 11.11, *p* < 0.01] in both groups.

### Instruments

*Word reading and pseudoword reading* were evaluated through the reading of 42 words and 42 pseudowords, respectively. Words were of different frequency, length, and orthographic complexity. Pseudowords were of different length and orthographic complexity. The total scores were, on the one hand, the sum of the scores achieved in word reading and, on the other, the sum of the scores achieved in pseudoword reading, when both types of word (words and pseudowords) were read accurately and/or fluently (up to a maximum of two points per item: one point for hesitant reading, two points for accurate and non-hesitant reading). For children who spoke Spanish, the Word Reading and Pseudoword Reading tests from the Spanish Reading and Writing Test (LEE) were used (Defior et al., 2006). The internal consistency reliability for the word and pseudoword reading tests among Year One pupils using Cronbach's alpha statistics were *α* = 0.80 and *α* = 0.82, respectively. The study of convergent validity also yielded satisfactory indicators, finding positive and significant correlations between the LEE test and the PROLEC test (Cuetos et al., 1996) for word reading (*r* = 0.51, *p* < 0.01) and pseudoword reading (*r* = 0.32, *p* < 0.01). The PROLEC test evaluates reading processes in school children aged 6 to 9, through different tests such as word and pseudoword reading. The word reading test evaluates the capacity to recognize words of different frequency, length, and syllable structure, and the pseudoword reading test evaluates the capacity to recognize non-words of the same length and syllable structure. For the Arabic-speaking children, the same number of words was used as in Spanish, using Arabic words used frequently in children’s language and of the same length (number of syllables), the same morphology (noun, adjective, verb), and the same frequency (low, medium, high) (Tunes, 1989) as those used in Spanish. The same number of pseudowords was also used, using words in Arabic of the same length as the pseudowords used in Spanish, and changing one letter. The words and pseudowords used in Arabic for the reading evaluation are listed in the “Appendix”. The validity of these tests is adequate according to the statistical tests carried out (Hutchenson & Sofroniou, 1999; Kline, 1999). Both tests present a one-dimensional structure and a saturation index greater than 0.49 and 0.36, explained variance of 44.07% and 38.97%, and a sampling adequacy index of 0.89 and 0.86, respectively. All the items present a homogeneity index of 0.33 and 0.35, and adequate internal consistency of 0.95 and 0.94, respectively.

*Knowledge of letters* was evaluated through the recognition of all the letters in each language, with the total score given by the number of letters read correctly. The children had to say the name or sound of each letter. For the Spanish-speaking children, the Knowledge of letters exercise from the Spanish Reading and Writing Test (LEE) was used (Defior et al., 2006). The internal consistency obtained for this test according to Cronbach's alpha statistic was 0.60. For the Arabic-speaking children, all the letters in that language were used. All the Arabic letters used for reading are listed in the “Appendix”. Cronbach's alpha statistic yielded a very similar value to the previous one (α = 0.58).

*Phonological awareness* was evaluated through 14 words, in which the children had to isolate the sounds that make up the words presented orally, naming the different phonemes that comprised each word. The total score was the total number of phonemes they identified correctly. For the Spanish-speaking children, the Phonological awareness exercise from the Spanish Reading and Writing test (LEE) was used (Defior et al., 2006). Cronbach's alpha statistic indicates an internal consistency of 0.91. For the Arabic-speaking children, the same number of words was used that had the same length (number of letters), the same morphology (noun, adjective, verb), and the same frequency (low, medium, and high) (Tunes, 1989). The “Appendix” indicates the words used in Arabic to measure phonological awareness. Cronbach's alpha statistic indicated a reliability of 0.84.

*Rapid automatized naming* was evaluated in both groups using the Rapid Automatized Naming (RAN) test (Wolf & Denckla, 2003), as adapted by Gómez-Velázquez et al. (2010). It entails naming 200 visual stimuli classified equally in terms of letters, numbers, objects, and colors. The total score in alphanumerical rapid naming was the time taken to name the letters and numbers, and for non-alphanumerical rapid naming it was the time taken to name objects and colors (Bowey et al., 2005; González-Valenzuela et al., 2016). For the Arabic-speaking children, letters in Arabic were used, and the order in which the equivalent letters appear in the Spanish version was respected. Cronbach’s alpha statistic confirmed the reliability of the test (*α* = 0.79). The other components of the test were the same in Spanish and Arabic.

*Phonological Memory* was evaluated in both groups using the *Phonological Short-Term Memory* (PSTM) test developed by Soriano and Miranda (2010), based on the Hebrew phonological memory task (Geva et al., 2000). The test involved the oral repetition of a list of 20 pseudowords of different length (words in Latin that did not coincide with Spanish or Arabic lexicon and that do not have any similarity with the morphology of both languages). The pseudowords used were unknown in Spanish and Arabic and were pronounced the same way in both languages (for example: baculum/, conspicio/, devinco/) and the children had to repeat them orally. The total score for this test was obtained by adding together all the words repeated correctly. Cronbach’s alpha statistic for this test was 0.74.

### Procedure

Authorization to conduct the experiment was obtained from the University of Málaga’s Ethical Experimentation Committee (CEUMA), having previously gained informed consent from the parents or legal guardians of the selected pupils.

The selected tests were administered individually to each pupil by the authors of this paper over two sessions. In the first of them, the knowledge of letters, and the word and pseudoword reading tests were administered. In the second session, the phonological memory, phonological awareness, and rapid automatized naming (alphanumerical and non-alphanumerical) tests were administered.

### Statistical Analysis

First, to analyze the bivariate relationship between all the variables, Pearson’s correlation coefficients and their corresponding significance tests were calculated, having first verified the supposed parameters of normality, homoscedasticity, and linear relationship between the variables.

Then, in accordance with the principal objective of this study, multivariate regression analyses were conducted for explanatory purposes. Hence, to analyze the joint and individual effect of the cognitive variables on the reading variables, multivariate regressions were estimated for word and pseudoword reading, in each of the languages studied. For these analyses, cognitive variables were inserted that, in the bivariate analysis, had a probability associated to Pearson’s *r* statistic of less than 0.05.

The most parsimonious model with the best fit for each dependent variable was selected using a backward procedure guided by the researcher (Kleinbaum et al., 1988; Losilla et al., 2005). A maximum model was used with the potential explanatory variables and first order terms of interaction detected in the previous bivariate analysis, in the order established by the researcher. Based on this first model, in a sequential process, the statistically non-significant regressor in each step was eliminated, beginning with the effects of interaction, and continuing with the principal effects, according to the regression coefficients, their statistical significance, the accuracy (amplitude) of their confidence intervals, and their standard error. With the variables retained, the final model was constructed. The overall significance was evaluated using Fisher’s *F* test, and the statistical significance of the model’s parameters was evaluated using Student’s *t* test (two-tailed). The total variance of each dependent variable attributable to the regression model was evaluated using the coefficient of determination ($${R}^{2})$$ and the adjusted coefficient of determination ($${\overline{R} }^{2}$$). The contribution made by each explanatory variable to the total variance of the dependent variable was evaluated using the semi-partial correlation coefficient ($${sr}_{i}^{2}$$). The fit of the data to the assumptions of the linear regression model was verified a posteriori using residual diagnostics. Evaluation of multicollinearity, carried out using the variance inflation factor (VIF), yielded values smaller than 10, indicating that multicollinearity here was not severe.

Data processing and statistical analysis were carried out using version 24 of the Statistical Package for the Social Sciences (SPSS).

## Results

The results of Pearson’s correlations between all the variables included in this study are given in Table 2 for each group of children in the different languages.

**Table 2:** Descriptive statistics and correlations separately by group

Variables	Range	1	2	3	4	5	6	7	
*Spanish Language (n = 128)*									
1. WR	6–76	–							
2. PSWR	9–69	.90**	–						
3. PA	0–12	.61**	.65**	–					
4. PM	6–20	.43**	.37**	.32**	–				
5. KL	9–29	.54**	.52**	.55**	.24**	–			
6. ARN	56–189	− .30**	− .31**	− .13	− .13	− .15	–		
7. NARN	120–401	− .00	− .01	− .06	− .01	− .13	.10	–	
*Arabic Language* (*n* = 113)									
1. WR	8–84	–							
2. PSWR	5–84	.85**	–						
3. PA	1–14	.20**	.21*	–					
4. PM	5–20	.15	.17	.03	–				
5. KL	17–28	.48**	.40**	.09	.24**	–			
6. ARN	72–228	− .17	− .10**	.13	− .13	− .22*	–	–	
7. NARN	139–840	− .00	− .01	.24*	− .01	− .28**	.45**	.45**	

^**^Pearson *r* correlation coefficient significant at *p* < .01; *Pearson *r* correlation coefficient significant at *p* < .05

*SD*  standard deviation, *WR* word reading (n° of correct responses), *PSWR* pseudoword reading (nº of correct responses), *PA* phonemic awareness (nº of correct responses), *PM* phonological memory (nº of correct responses), *KL* knowledge of letters (nº of correct responses), *ARN* alphanumeric rapid naming (seconds), *NARN* non-alphanumeric rapid naming (seconds)

In the group of Spanish-speaking children, following bivariate analysis, statistically significant relationships were found between word reading and pseudoword reading, and the variables phonological awareness, knowledge of letters, phonological memory, and alphanumerical rapid naming, in that order, according to the magnitude of the relationship. However, no relationship was found between the two reading variables and non-alphanumerical rapid naming.

Regarding the relationship between the cognitive variables, a significant relationship was found between phonological awareness and knowledge of letters, phonological awareness and phonological memory, as well as between knowledge of letters and phonological memory. No statistically significant relationship was found between any of them and alphanumerical or non-alphanumerical rapid naming, or between these two.

In the group of Arabic-speaking children, statistically significant relationships were found between word reading and the cognitive variables knowledge of letters and phonological awareness, as well as between pseudoword reading and knowledge of letters, phonological awareness, and alphanumerical rapid naming, in that order, according to the size of the correlation coefficient. However, no relationship was found in any case with the cognitive variables phonological memory and non-alphanumerical rapid naming, and no relationship was found in any case with the cognitive variables phonological memory and non-alphanumerical rapid naming.

Among the cognitive variables, a statistically significant relationship was found between phonological awareness and non-alphanumerical rapid naming, between phonological memory and knowledge of letters, between knowledge of letters and non-alphanumerical rapid naming, between knowledge of letters and alphanumerical rapid naming, as well as between alphanumerical rapid naming and non-alphanumerical rapid naming.

Below, we analyze how cognitive abilities explained reading in each language. To this end, hierarchical regressions were carried out, including the cognitive variables as the independent variables and the interactions between them that had correlated significantly in the previous bivariate analysis. Word reading (Table 3) and pseudoword reading (Table 4) were included as the dependent variables, for each language.

**Table 3:** Multivariate regression analysis for word reading in Spanish and Arabic language

	Variables	*B*	*SE*	*β*	*t*	*p*	*sr*	*VIF*
*WR*								
Spanish language (n = 128)								
	Intercept	26.29	36.27		0.79	.431		
Model 1	PA	0.51	2.61	.11	0.19	.845	.01	80.91
	PM	− 0.66	2.29	− 0.13	− 0.29	.772	− .02	49.03
	KL	0.07	1.53	0.02	0.05	.961	.00	48.50
	ARN	− 0.08	0.03	− 0.16	− 2.46	.015	− .15	1.11
	PA × PM	0.10	0.11	0.41	0.91	.364	.06	52.12
	PA × KL	− 0.02	0.08	− 0.11	− 0.22	.821	− .01	63.06
	KL × PM	0.05	0.10	0.40	0.55	.581	.03	135.31
	^a^ *F* (7, 120) = 19.05, *p* = .000; R^2^ = 0.52; R^2^ adjusted = 0.50; R = 0.72							
	Intercept	0.87	8.22		0.10	.916		
Model 2	PA	1.76	0.35	0.38	4.95	.000	.31	1.51
	PM	1.16	0.34	0.22	3.34	.001	.21	1.13
	KL	0.87	0.26	0.25	3.31	.001	.21	1.45
	ARN	− 0.09	0.03	− 0.18	− 2.86	.005	− .18	1.03
	^a^* F* (4, 123) = 33.28, *p* = .000; R^2^ = 0.52; R^2^ adjusted = 0.50; R = 0.72							
*Arabic language (n* = *113)*								
	Intercept	− 84.40	23.38		− 3.61	.000		
Model 1								
	KL	4.83	0.86	0.46	5.63	.000	.46	1.01
	PA	0.73	0.38	0.16	1.90	.060	.15	1.01
	^a^* F* (2, 110) = 18.82, *p* = .000; R^2^ = 0.25; R^2^ adjusted = 0.24; R = 0.50							
Model 2								
	Intercept	− 81.52	23.60		− 3.45	.001		
	KL	4.98	0.86	0.48	5.76	.000	.48	1.00
	^a^* F* (1, 111) = 33.26, *p* = .000; R^2^ = 0.23; R = 0.48							

*WR* word reading, *PA* phonemic awareness, *PM* phonological memory, *KL* knowledge of letters, *ARN* alphanumeric rapid naming, *SE* standard error, *sr* semi-partial correlation coefficient, *VIF* variance inflation factor

^a^Goodness-of-fit tests for multivariate regression models: Global test F; coefficient of determination, R^2^; adjusted coefficient of determination, adjusted R^2^

**Table 4:** Multivariate regression analysis for pseudoword reading in Spanish and Arabic language

	Variables	*B*	*SE*	*β*	*t*	*p*	*sr*	*VIF*
*PSWR*								
*Spanish language (n* = *128)*								
	Intercept	3.23	30.36		0.10	.916		
Model 1	PA	0.04	2.39	0.01	0.99	.988	.00	80.91
	PM	1.61	2.10	0.34	0.44	.442	.07	49.03
	KL	0.83	1.40	0.26	0.55	.552	.05	48.50
	ARN	− 0.08	0.03	− 0.19	0.00	.005	− .25	1.11
	PA × PM	− 0.00	0.10	− 0.02	0.95	.955	− .00	52.12
	PA × KL	0.08	0.07	0.57	0.25	.256	.10	63.06
	KL × PM	− 0.04	0.09	− 0.29	0.68	.686	− .03	135.31
	^a^ *F* (7, 120) = 19.28, *p* = .000; R^2^ = 0.53; R^2^ adjusted = 0.50; R = 0.73							
	Intercept	8.26	7.49		1.10	.272		
Model 2	PA	1.96	0.32	0.46	6.04	.000	.37	1.51
	PM	0.72	0.32	0.15	2.27	.025	.14	1.13
	KL	0.65	0.24	0.20	2.72	.008	.17	1.45
	ARN	− 0.09	0.03	− 0.19	− 3.09	.002	− .19	1.03
	^a^* F* (4, 123) = 33.88, *p* = .000; R^2^ = 0.52; R^2^ adjusted = 0.51; R = 0.72							
	Intercept	18.21	6.17		2.95	.004		
Model 3	PA	2.13	0.32	0.50	6.65	.000	.42	1.43
	ARN	− 0.09	0.03	− 0.21	− 3.26	.001	− .20	1.03
	KL	0.70	0.24	0.21	2.83	.005	.18	1.44
	^a^* F* (3, 124) = 42.06, *p* = .000; R^2^ = 0.50; R^2^ adjusted = 0.49; R = 0.71							
*Arabic language (n* = *113)*								
	Intercept	− 19.75	18.88		− 1.04	.298		
Model 1	ARN	− 0.10	0.03	− 0.31	− 3.61	.000	− .31	1.05
	KL	1.89	0.65	0.25	2.92	.004	.25	1.05
	^a^ *F* (2, 110) = 13.73, *p* = .000; R^2^ = 0.20; R^2^ adjusted = 0.18; R = 0.45							

*PSWR* pseudowords reading, *PA* phonemic awareness, *PM* phonological memory, *KL* knowledge of letters, *ARN* alphanumeric rapid naming, *SE* = standard error, *sr* semi-partial correlation coefficient, *VIF* variance inflation factor

^a^Goodness-of-fit tests for multivariate regression models: Global test F; coefficient of determination, R^2^; adjusted coefficient of determination, adjusted R^2^

Using the first estimated model, the independent variables were eliminated one by one in the order determined by the researcher. In general, all the models for each dependent variable, and for each language, were statistically significant, and no final model included the effects of interaction inserted in the initial model. Below we describe the results obtained for word reading and pseudoword reading in each language considered in the study, according to the final models selected, having verified by means of residual analysis that the data presented a good fit to the assumptions of the linear regression model (see Tables 3 and 4).

### Word Reading

To study the individual and joint contribution of phonological awareness, phonological memory, knowledge of letters, and alphanumerical rapid naming to word reading among the Spanish-speaking group of children, whilst controlling for possible interactions between the cognitive variables (see Table 3), a final model was obtained, in two stages, with a good overall fit [*F*(4, 123) = 33.28, *p* < 0.001], which included the explanatory variables phonological awareness [*t*(127) = 4.95, *p* < 0.001], phonological memory [*t*(127) = 3.34, *p* < 0.01], knowledge of letters [*t*(127) = 3.31, *p* < 0.01] and alphanumerical rapid naming [*t*(127) = -2.86, *p* < 0.01]. The coefficient of determination (${\overline{R} }^{2}$ adjusted = 0.50) indicated that 50% of the variance of the response variable is explained by the regression model in which, in order of importance, phonological awareness contributes 9.61%, phonological memory 4.41%, knowledge of letters 4.41%, and alphanumerical rapid naming 3.24%, according to their corresponding semi-partial correlation coefficient (${sr}_{i}^{2}$).

Equally, to study the contribution made by the cognitive variables knowledge of letters and phonological awareness to word reading among Arabic-speaking children (see Table 3), the final estimated model obtained, also in two steps [*F*(1, 111) = 33.26, *p* < 0.001], included only the first variable, knowledge of letters [*t*(127) = 5.76, *p* < 0.001], explaining 23% of the variance of the response variable according to the coefficient of determination (*R*^2^ = 0.23).

### Pseudoword reading

Table 4 summarizes the individual and joint contribution made by the variables phonological awareness, phonological memory, knowledge of letters and alphanumerical rapid naming to pseudoword reading among Spanish-speaking children, controlling for possible interactions between significantly related cognitive variables. In three steps, a good final model was obtained [*F*(3, 124) = 42.06, *p* < 0.001] with the cognitive variables phonological awareness [*t*(127) = 6.65, *p* < 0.001], alphanumerical rapid naming [*t*(127) = − 3.26, *p* < 0.01] and knowledge of letters [*t*(127) = 2.83, *p* < 0.01]. According to the coefficient of determination (${\overline{R} }^{2}$ adjusted = 0.49), 49% of variance in pseudoword reading is explained by the regression model, in which, in order of importance, according to the corresponding semi-partial correlation coefficient (${sr}_{i}^{2}$), phonological awareness contributes 17.64%, alphanumerical rapid naming 4%, and knowledge of letters 3.24%.

Similarly, for the study of the contribution made by alphanumerical rapid naming and knowledge of letters to pseudoword reading among Arabic-speaking children (see Table 4), in just one step a significant final model was obtained [*F*(2, 110) = 13.73, *p* < 0.001] that included both cognitive variables: alphanumerical rapid naming [*t*(127) =  − 3.61, *p* < 0.001] and knowledge of letters [*t*(127) = 2.92, *p* < 0.01]. The coefficient of determination (${\overline{R} }^{2}$ adjusted = 0.18) indicated that 18% of variance in the variable pseudoword reading is explained by the regression model, in which alphanumerical rapid naming contributes 9.61% and knowledge of letters 6.25%, according to the magnitude of the corresponding semi-partial correlation coefficient (${sr}_{i}^{2}$).

## Discussion

This study analyzed the degree to which certain cognitive variables (knowledge of letters, phonological awareness, rapid automatized naming, and phonological memory) contributed to explaining word and pseudoword reading among children in the first year of Primary Education in two languages of different orthographic consistency (Spanish and Arabic).

The findings highlight, on the one hand, that in Spanish, phonological awareness, knowledge of letters, and alphanumerical rapid naming contribute to explaining word and pseudoword reading, and that phonological memory is also related to word reading. On the other hand, in Arabic, knowledge of letters contributes to explaining word and pseudoword reading, and alphanumerical rapid naming is also a strong predictor in the case of pseudoword reading. In other words, in both measures of reading, phonological awareness, knowledge of letters, and alphanumerical rapid naming are strong predictors in Spanish, and knowledge of letters and alphanumerical rapid naming are strong predictors in Arabic. In word and pseudoword reading in both languages, phonological memory presents a weak or null contribution, and non-alphanumerical rapid naming does not contribute to the explanation in any case.

These results indicate, first, that the cognitive variables analyzed contribute similarly to explaining the different measures of reading—words and pseudowords—in each of the groups of six-year-olds, when the children have been taught to read. In both types of reading measures, there do not appear to be great differences between the two languages with regard to the predictors considered, according to the specific contribution made by each of them, which is similar in each reading measure in both groups. These results could be explained by different types of orthographic representations being grounded in the repeated use of phonological representations and in grapheme-phoneme translation (Share, 2008; Share & Shalev, 2004) or in the access to the visual representations that make up the orthographic lexicon, according to the different writing systems and their grapheme-sound consistency in the explanation of the reading (Daniels & Share, 2018).

Second, the results indicate that the cognitive variables analyzed explain differently the different measures of reading in each group at this age, although they present certain similarities. In both groups, there appear to be some differences in each measure of reading with regard to the predictors considered. Namely, the predictive nature of the variables analyzed is different in each group in both measures of reading, as will be commented below. Similar results are found in other studies that defend the predictive variability of some of these phonological processes according to orthographic consistency (Elheberi et al., 2011; Furness & Samuelsson, 2009; Georgiou et al., 2008; Layes et al., 2015). These results, therefore, endorse the importance of phonological processing in languages of different consistency, although they highlight that not all the factors analyzed have the same relevance at the start of schooling in each language. This would support the theory of granularity, insofar as the degree of orthographic consistency can influence the contributions of these cognitive variables on the acquisition of reading skill (Carrillo et al., 2013; Ziegler & Goswami, 2005).

As for knowledge of letters, the findings support those reported in studies that argue the fundamental role played by this skill in different languages, such as Spanish and Arabic, given that it enables decoding (Assad & Eviatar, 2014; Caravolas et al., 2012; Landerl et al., 2019; Tolchinsky et al., 2012). It should also be noted that the role of knowledge of letters is more relevant in the case of Arabic, with greater percentages of explained variance in this case. This is in line with studies that highlight the relevance of knowledge of letters in different languages, but even more so in less consistent languages because they demand greater grapheme-phoneme conversion analysis (Caravolas et al., 2012, 2013). In less consistent languages, such as English, knowledge of letters can be important because it provides children with an identifiable reference to associate with phonemes, and because it reflects precision in the representation of individual letters, which is necessary for the accurate recognition of words (Georgiou et al., 2012). In contrast, in highly consistent languages, such as Finnish, all letter names provide the sound of the letter, which is independent of the context in which it appears. Therefore, knowledge of the names of letters and sounds, and phonological assembly, are requirements for adequate decoding (Georgiou et al., 2012). Therefore, evaluation of the knowledge of letters is important for detecting possible reading difficulties as have been done in some studies (Landerl et al., 2019; Vaessen & Blomert, 2013).

As for phonological awareness, the results indicate that this variable only contributes to explaining the different measures of reading in the case of Spanish and does not contribute in the case of Arabic. The importance of phonological awareness in Spanish is also confirmed by other studies that demonstrate the importance of this process in grapheme-phoneme conversion at different ages and in different measures of reading in this language (Aguilar-Villagrán et al., 2010; González-Valenzuela et al., 2016; Suárez-Coalla et al., 2013; Tolchinsky et al., 2012). Regarding the relationship found between phonological awareness and reading in Arabic, this is in line with other findings that highlight that this type of measure at a young age is not as important as it is in more consistent languages, given the importance of visual processing variables compared to phonological variables in this opaque language (Elheberi & Everatt, 2007; Elheberi et al., 2011; Layes et al., 2015). For example, in Arabic, some letters have more than one written form. The form of the letters depends on their placement within the word. Some have dots in different positions, others do not, some graphemes are written but not pronounced, or are pronounced differently to the way they are written (diglossia) (Abu-Rabia & Taha, 2004; Abu-Rabia et al., 2003; Taha, 2013; Taha et al., 2013). Arabic is, therefore, a less consistent, more opaque language than Spanish, as we can see from some of the characteristics indicated above. This would support the theory of granularity and the idea that the degree of consistency is relative, depending on the orthographic systems being compared (Carrillo et al., 2013). The lower the orthographic consistency, the less accurate the representation of the graphemes on account of their phonological structure (coarse grain) (Ziegler & Goswami, 2005). On the other hand, however, the current results contrast studies that consider phonological awareness to be more relevant among less consistent languages than in more consistent ones since more consistent languages demand less grapheme-phoneme conversion (Georgiou et al., 2008; Vaessen et al., 2010; Ziegler et al., 2010). In the most consistent languages, phonological knowledge can be a predictor only in the first years of literacy (Aarnoutse et al., 2005), since the effect of the correspondences between grapheme and sound is powerful enough to ensure the children's phonological recoding skills after a few months of experience, regardless of their levels of phonological awareness prior to reading (Caravolas, 2006; Georgiou et al., 2008).

Furthermore, the results also differ from those found in studies that signal the importance of phonological awareness in Arabic as well (Abu-Ahmad et al., 2014; Assad & Eviatar, 2014; Assadi et al., 2017; Tibi & Kirby, 2018). It should be noted that these studies were conducted on older children with greater reading experience, and this might be a factor in explaining the differences encountered. Based on these results, one practical implication could be that phonological awareness should be an important component of reading instruction in languages of different consistency, although in opaque languages it would be more relevant for slightly older learners.

In relation to rapid automatized naming, the results show that the non-alphanumerical component (colors and objects) does not contribute to explaining reading in either of the languages considered at this age. Some studies have also highlighted the scant importance of this component in different languages in terms of reading speed or fluency, attributing this to its closer relationship with visual processing than phonological processing (Aguilar-Villagrán et al., 2010; Bowey et al., 2005; Georgiou et al., 2008; González-Valenzuela et al., 2016). However, the alphanumerical component does contribute to explaining reading in both languages at this age: in Spanish, in the case of word and pseudoword reading, and in Arabic, in pseudoword reading. In the case of pseudowords, the percentage of explained variance is higher in Arabic. These results concur with studies that argue the importance of this variable in languages of different orthographic consistency, as well as studies that highlight that alphanumerical automatized naming is more relevant in less consistent languages (Caravolas et al., 2012, 2013; Furnes & Samuelsson, 2010; Moll et al., 2014). However, they disagree with the findings of studies that state its greater importance in more consistent languages (Georgiou et al., 2008; Landerl et al., 2019; Ziegler et al., 2010). These differences could be due to the fact that, in some cases, only the naming of digits or measures of reading accuracy have been used, and also to the fact that the subjects were pre-readers. Another possible explanation is that the influence of alphanumerical automatized naming might depend on its relationship with phonological awareness in each language (Escribano & Katzir, 2008; González-Seíjas et al., 2013; Suárez-Coalla et al., 2013). It could be said that the predictive nature of alphanumerical naming not only depends on orthographic consistency, but also on the type of measure used and its relationship with phonological awareness in each language. One practical implication of these results would be that, in order to detect reading disabilities in different languages, and at the start of compulsory schooling, alphanumerical rapid naming should be used, but not non-alphanumerical rapid naming.

With regard to phonological memory, the results indicate that it only contributes to explaining word reading in Spanish. These results coincide with studies conducted in different languages and for different ages, which establish a weak or null relationship between phonological memory and the different measures of reading. These studies consider that this weak relationship only appears when rapid naming and phonological awareness are considered jointly (Georgiou et al., 2008; González-Valenzuela et al., 2016; Parrila et al., 2004; Suárez-Coalla et al., 2013), as is the case in Spanish, but not in Arabic. One particularly striking observation is that phonological memory does not contribute to word and pseudoword reading in Arabic. This could be because the pseudowords considered have a similar structure to Spanish, in contrast to Arabic, although they are both pronounced in the same way in both groups. It may also be due to the ability to maintain phonological representations of words in short-term memory that is related to language learning (Sari et al., 2020), but not to reading acquisition. These results suggest that, in practice, phonological memory might not be the most relevant component in detecting reading disabilities in different languages at this age.

In short, the results found largely support the findings of studies that argue the importance of phonological processing in languages with different orthographic consistency and the predictive variability of some of these processes in explaining different measures of reading at six years of age. This has important practical implications for reading instruction, since it suggests that teaching methods that incorporate phonological components would be advisable in languages with different consistency.

These results should be approached with caution, since we were not able to control for the type of reading instruction given in both groups, and the sample was only made up of pupils in the first year of Primary Education. Furthermore, this study has not considered the influence of the vowelization effect (use of diacritical marks to represent short vowels) in Arabic, since these characteristics are not present in the Spanish alphabet.

In future studies, it would be advisable to analyze the effects of instruction in phonological processing on reading performance among Arabic-speaking children, to discern its influence in such an inconsistent language. It would also be important to analyze whether the results found are maintained over time or whether, on the contrary, at later ages, when children have more reading experience in both languages, the role of each of the variables considered is similar or different.
